# The Role of Natural Products in Targeting Cardiovascular Diseases via Nrf2 Pathway: Novel Molecular Mechanisms and Therapeutic Approaches

**DOI:** 10.3389/fphar.2018.01308

**Published:** 2018-11-15

**Authors:** Bee Kee Ooi, Kok-Gan Chan, Bey Hing Goh, Wei Hsum Yap

**Affiliations:** ^1^School of Biosciences, Taylor’s University, Subang Jaya, Malaysia; ^2^Division of Genetics and Molecular Biology, Institute of Biological Sciences, Faculty of Science, University of Malaya, Kuala Lumpur, Malaysia; ^3^International Genome Centre, Jiangsu University, Zhenjiang, China; ^4^Biofunctional Molecule Exploratory Research Group, School of Pharmacy, Monash University Malaysia, Bandar Sunway, Malaysia; ^5^Novel Bacteria and Drug Discovery Research Group, School of Pharmacy, Monash University Malaysia, Bandar Sunway, Malaysia; ^6^Asian Centre for Evidence Synthesis in Population, Implementation and Clinical Outcomes, Health and Well-Being Cluster, Global Asia in the 21st Century Platform, Monash University Malaysia, Bandar Sunway, Malaysia; ^7^Center of Health Outcomes Research and Therapeutic Safety, School of Pharmaceutical Sciences, University of Phayao, Phayao, Thailand

**Keywords:** nuclear factor erythroid 2-related factor 2 (Nrf2), cardiovascular diseases (CVDs), natural products, oxidative stress, nuclear factor-κB (NF-κB)

## Abstract

Cardiovascular diseases (CVDs) are closely linked to cellular oxidative stress and inflammation. This may be resulted from the imbalance generation of reactive oxygen species and its role in promoting inflammation, thereby contributing to endothelial dysfunction and cardiovascular complications. Nuclear factor erythroid 2-related factor 2 (Nrf2) is a transcription factor that plays a significant role in regulating expression of antioxidant and cytoprotective enzymes in response to oxidative stress. Natural products have emerged as a potential source of bioactive compounds which have shown to protect against atherogenesis development by activating Nrf2 signaling. This review aims to provide a comprehensive summary of the published data on the function, regulation and activation of Nrf2 as well as the molecular mechanisms of natural products in regulating Nrf2 signaling. The beneficial effects of using natural bioactive compounds as a promising therapeutic approach for the prevention and treatment of CVDs are reviewed.

## Introduction

Cardiovascular disease (CVD) is a major health complication which accounts for 15.2 million deaths worldwide in 2016 (World Health Organization, 2018). Atherosclerosis, as characterized by the formation of plaques with bulks of modified low density lipoprotein (LDL), immune cells, smooth muscle cells and cellular debris in the arterial intima, is the primary cause of CVD. The molecular mechanisms underlying CVD have been extensively investigated over the past decades. It has been demonstrated that the involvement of oxidative stress and inflammation are associated with the pathogenesis of CVD. Oxidative stress which results from excessive generation of reactive oxygen species/reactive nitrogen species (ROS/RNS) can trigger inflammation, which contribute to LDL oxidation, endothelial dysfunction, atherosclerotic plaque formation, plaque rupture, vascular remodeling, and atherothrombosis (Pashkow, 2011; Hajjar and Gotto, 2013; Hussain et al., 2016). In response to increased ROS/RNS levels under oxidative stress condition, the cells will induce the expression of antioxidant proteins and phase II detoxification enzymes such as heme oxygenase 1 (HO-1), aldo-keto reductase (AKR), peroxiredoxin 1 (PRX), γ-glutamyl cysteine ligase (γ-GCL) glutamate-cysteine ligase modifier subunit (GCLM), superoxide dismutase (SOD), NADPH quinine oxidoreductase 1 (NQO1) and others (Menegon et al., 2016; Jeddi et al., 2017). Transcriptional regulation of these enzymes is mainly controlled by nuclear factor erythroid 2-related factor 2 (Nrf2), a transcription factor that plays as a central role in intracellular redox homeostasis. In addition, Nrf2 also protects against macrophage foam cells formation by regulating expression of scavenger receptors, ATP-binding cassette (ABC) transporters, and multidrug resistance-associated proteins (MRPs) (Jeddi et al., 2017; Ooi et al., 2017). For instance, deficiency of Nrf2 in the bone marrow has been shown to aggravate atherosclerosis in LDL receptor-null (LDLR^-/-^) mice (Collins et al., 2012; Ruotsalainen et al., 2013). These evidences support the notion that Nrf2 protects against atherosclerosis. Although some studies showed that Nrf2 exhibits pro-atherogenic effects, its molecular mechanisms remain unclear (Sussan et al., 2008; Barajas et al., 2011; Freigang et al., 2011; Harada et al., 2012; Ruotsalainen et al., 2018).

**FIGURE 1:**
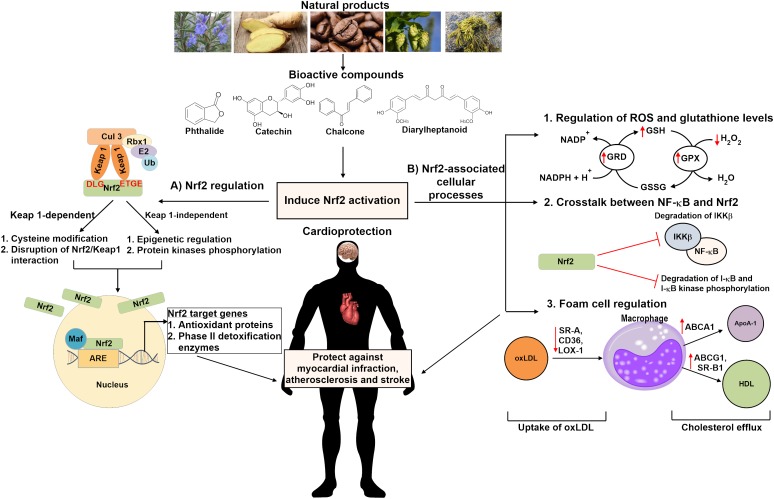
Schematic overview of the cardioprotective mechanisms of bioactive compounds derived from natural products in activating Nrf2 signaling pathway. Natural compounds may induce Nrf2 activation via the (A) Keap1-dependent or Keap1-independent pathway which involves cysteine modification, disruption of Nrf2/Keap1 interaction, epigenetic regulation and/or protein kinases phosphorylation. Nrf2 activation might also be induced through (B) Nrf2-associated cellular processes, including regulation of ROS and glutathione levels, inhibiting NF-κB inflammatory signaling pathway as well as controlling oxLDL uptake and cholesterol efflux in foam cells regulation. These bioactive compounds protect against cardiovascular diseases such as stroke, atherosclerosis and myocardial infraction. Nrf2 indicates nuclear factor erythroid 2-related factor 2; Keap 1, Kelch-like ECH-associated protein 1; Ub, ubiquitin; Maf, musculoaponeurotic fibrosarcoma; ARE, antioxidant response element; ROS, reactive oxygen species; GRD, glutathione reductase; GPX, glutathione peroxidase; GSH, glutathione; GSSG, glutathione disulfide; NF-κB, nuclear factor kappa-B kinase; IKK-β, inhibitor of nuclear factor kappa-B kinase subunit beta; oxLDL, oxidized low density lipoprotein; SR-A, scavenger receptor class A; CD36, scavenger receptor class B; LOX-1, lectin-type oxidized LDL receptor 1; ABC, ATP-binding cassette transporter; SR-B1, scavenger receptor class B type 1; ApoA-1, apolipoprotein A-1; HDL, high density lipoprotein.

Natural products offer unique structural and chemical diversity that serve as a source of novel drug leads and therapeutic agents. Natural products have been shown to alleviate oxidative stress-induced diseases such as CVD, neurodegenerative diseases, cancer and metabolic disorders by regulating the Nrf2/antioxidant responsive element (ARE) pathway (Waltenberger et al., 2016; Basak et al., 2017; Matzinger et al., 2017). Natural products derived from olive oil (hydroxytyrosol) and red wine (resveratrol) have been demonstrated to inhibit ROS production. Meanwhile, both bioactive compounds have also been reported to enhance Nrf2 nuclear translocation and decrease miRNA-146a expression, a pro-inflammatory marker (Bigagli et al., 2017). Recent studies revealed that sulforaphane, an isothiocyanate derived from cruciferous vegetables, protects against CVD due to its antioxidant and anti-inflammatory effects mediated through the Nrf2 signaling pathway (Bai et al., 2015). These evidences suggested that natural products may serve as a promising therapeutic approach for the prevention and treatment of CVD associated with oxidative stress. This review will discuss on the current knowledge on the molecular mechanisms of cardioprotective bioactive compounds targeting the Nrf2/ARE signaling pathway (Figure 1).

## Regulation of Nrf2 Signaling Pathway

### Structural Features of Nrf2

**FIGURE 2:**
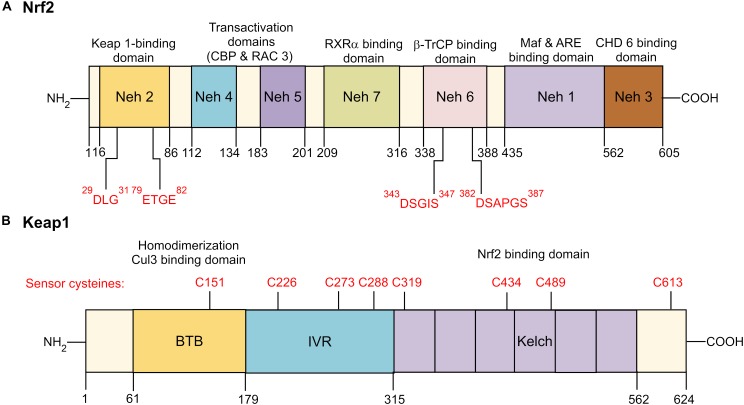
Domain structure of Nrf2 and Keap1. **(A)** Functional Nrf2-ECH homology (Neh) domains: Neh 1 is the binding site for small Maf proteins and ARE. Neh 2 serves as the binding site for Keap1 by interacting with low-affinity DLG and the high affinity ETGE motifs. Neh 3-5 are transactivation domains for Nrf2. Neh 6 is a serine-rich domain that negatively controls the Nrf2 stability by β-TrCP interacts with DSGIS and DSAPGS motifs. Neh 7 interacts with RXRα, a nuclear receptor responsible for suppression of Nrf2/ARE signaling pathway. **(B)** Functional Keap1 domains: N-terminal region, BTB dimerization domain, cysteine-rich IVR domain, six Kelch/DRG domain, and C-terminal region. BTB is responsible for Keap1 homodimerization and association with cullin (Cul3)-containing E3 ubiquitin ligase complex. IVR consists of reactive cysteine residues, including C226, C273 and C288. DRG domain is responsible for Nrf2 binding to DLG and ETGE motifs. Nrf2 indicates nuclear factor erythroid 2-related factor 2; Keap 1, Kelch-like ECH-associated protein 1; RXRα, retinoid X receptor α; β-TrCP, β-transducin repeat-containing protein; Maf, musculoaponeurotic fibrosarcoma; ARE, antioxidant response element; CHD 6, chromo-ATPase/helicase DNA-binding protein 6; BTB, Broad-Complex, Tramtrack, and Bric-a-Brac; IVR, intervening region; DRG, double glycine repeats.

Human Nrf2 (*NFE2L2*) protein comprises of 605 amino acid residues with molecular weight of 67.7 kDa (Cho, 2013). The Nrf2 gene consists of seven functional domains, also known as the Nrf2-ECH homology (Neh) domains (Namani et al., 2014; Canning et al., 2015). The position of each functional domain of Nrf2 is illustrated in Figure 2A. Neh 1 domain comprises of highly conserved basic region-leucine zipper (CNC-bZIP) region that dimerizes with small musculoaponeurotic fibrosarcoma (Maf) proteins and binds to ARE, a *cis*-acting enhancer sequence found in the promoter region of many genes encoding antioxidant and phase II detoxification enzymes or proteins. Neh 2 domain acts as a negative regulatory domain as it contains the two degrons, known as high-affinity ETGE motif and the lower-affinity DLG motif. These motifs specifically interact with Kelch-like ECH-associated protein 1 (Keap1) which mediate ubiquitination and degradation of Nrf2. The carboxy-terminal of Neh 3 domain is a transactivation domain that recruits the chromo-ATPase/helicase DNA-binding protein 6 (CHD 6) and drives ARE- gene expression. Both Neh 4 and Neh 5 also function as transactivation domains which are involved in the interaction with cAMP response element-binding protein (CREB)-binding protein (CBP) and receptor-associated coactivator 3 (RAC 3). Meanwhile, Neh 6 domain negatively regulates Nrf2 stability via glycogen synthase kinase-3 (GSK-3)/β-transducin repeat-containing protein (β-TrCP)-mediated degradation. It contains two highly conserved redox-independent degrons known as DSGIS and DSAPGS motifs. DSAPGS motif interacts with β-TrCP, which serves as a substrate receptor for the S-phase kinase-associated protein 1- Cullin 1- RING box protein-1/regulator of cullins-1 (Skp1–Cul1–Rbx1/Roc1) ubiquitin ligase complex. This results in ubiquitination and degradation of Nrf2 via the Keap1-independent pathway. Besides, suppression of Nrf2/ARE signaling pathway may be mediated via interacting with Neh 7 domain and the DNA-binding domain of retinoid X receptor α (RXRα).

### Regulation of Nrf2 Activity

#### Keap1-Dependent Regulation of Nrf2 Activity

**FIGURE 3:**
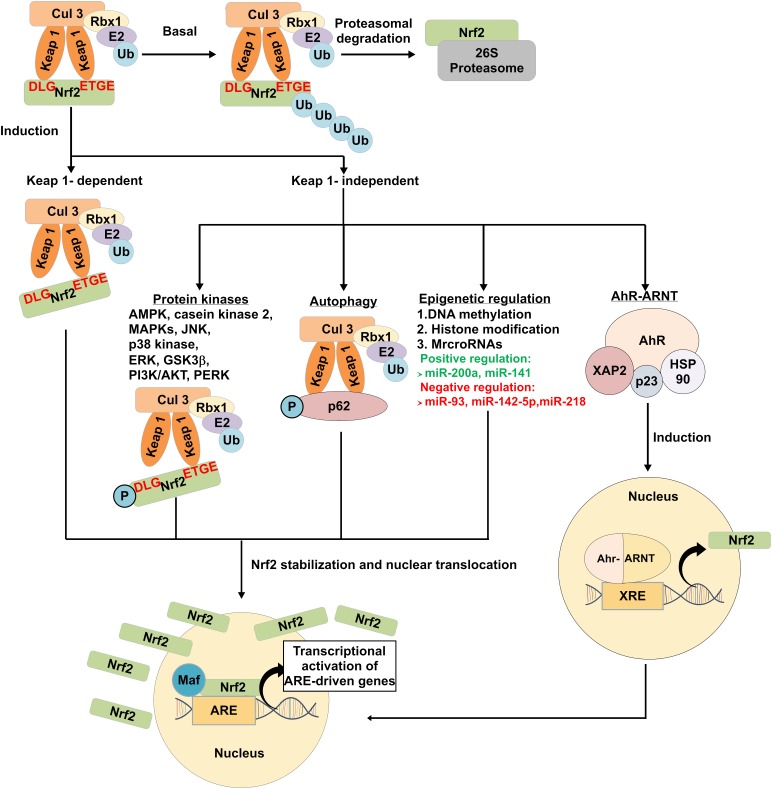
Modulation of Keap1-dependent and Keap1-independent mediated Nrf2 signaling pathways. Under basal condition, Nrf2 is constantly targeted by Keap1 for proteasomal degradation. Nrf2 may be induced by Keap1-dependent and Keap1-independent pathways. Under the Keap1-dependent pathway, exposure to oxidative stress and Nrf2 activators induce conformational change in the cysteine residues of Keap1, which disrupts the interaction between Kelch domain and DLG motif bound to Nrf2. This results in the release of Nrf2 from Keap1. Phosphorylation of protein kinases and autophagy adaptor proteins, epigenetic regulation and AhR-ARNT binding to XRE are associated with the release of Nrf2 in Keap1-independent-mediated pathways. Stabilized free cytosolic Nrf2 translocate into the nucleus, binds to ARE and induce transcription of antioxidant and detoxifying enzymes and proteins. Ub indicates ubiquitin; AMPK, AMP-activated protein kinase; MAPK, mitogen-activated protein kinase cascades; JNK, c-Jun N-terminal kinase; ERK, extracellular-signal-regulated kinase; GSK3β, glycogen synthase kinase-3β; PI3K/AKT, phosphatidylinositol-3-kinase; PERK, pancreatic endoplasmic reticulum kinase; p62, sequestosome-1; AhR-ARNT, aryl hydrocarbon receptor-aryl hydrocarbon receptor nuclear translocator; HSP90, heat shock protein 90; XAP2, X-associated protein 2; p23, HSP90 co-chaperone; ARE, antioxidant response element, and; XRE, xenobiotic response element.

Keap1 is a cysteine rich adaptor protein for cullin (Cul3)-containing E3 ubiquitin ligase complex which mediates Nrf2 ubiquitination and degradation by 26S proteasomes. It consists of five sub-sections (Figure 2B), namely the N-terminal region, BTB dimerization domain (Broad-Complex, Tramtrack, and Bric-a-Brac), cysteine-rich intervening (IVR) domain, six Kelch/double glycine repeats (DRG) domain, and C-terminal region (Namani et al., 2014; Basak et al., 2017). Under normal homeostatic condition, Nrf2 has a short half-life of approximately 20 min. They are maintained at low level and constantly targeted for proteasomal degradation (Kobayashi and Yamamoto, 2006). The Keap1-dependent Nrf2 regulatory pathway supports the notion that exposure to ROS or Nrf2 inducers such as epigallocatechin-3-gallate (EGCG), sulforaphane, dimethyl fumarate (DMF), and *tert*-butylhydroquinone (tBHQ) will result in conformational changes in Keap1 cysteine residues, which interferes the interaction between Kelch domain and DLG motif where the ETGE motif still bound to Nrf2. Consequently, Keap1 fails to align with the E2 ubiquitin-conjugating enzyme and thus Nrf2 are no longer targeted for ubiquitination and degradation. The accumulation of free cytosolic Nrf2 is translocated into the nucleus where it dimerizes with Maf protein and binds to ARE sequences, resulting in the expression of downstream target genes (Figure 3) (Basak et al., 2017; Matzinger et al., 2017; Ooi et al., 2017).

#### Keap1-Independent Regulation of Nrf2 Activity

Apart from Keap1-dependent mechanism, emerging bodies of evidences revealed that Nrf2 can also be regulated through a number of mechanisms independent of Keap1. These mechanisms include transcriptional and epigenetic regulation, autophagy and other signaling pathways (Figure 3).

##### Transcriptional regulation of Nrf2

Binding of Aryl hydrocarbon receptor-aryl hydrocarbon receptor nuclear translocator (AhR-ARNT) to xenobiotic response element (XRE) sequences is known to regulate Nrf2 activation. AhR is a member of the basic helix-loop-helix Per-ARNT-Sim (bHLH-PAS) family of transcription factors that functions as xenobiotic chemical sensor in eukaryotes (Furue et al., 2017; Nebert, 2017). The inactive form of AhR is stabilized in the cytoplasm in a complex form with heat shock protein 90 (HSP90), X-associated protein 2 (XAP2), and HSP90 co-chaperone p23 (Mimura and Fujii-Kuriyama, 2003; Quintana, 2013; Furue et al., 2017). Upon exposure to polycyclic aromatic hydrocarbon, AhR ligand complex translocate into the nucleus where it dissociates from HSP90 complex and dimerizes with ARNT and binds to XRE sequences at the promoter region and upregulates the expression of phase I and II metabolic enzymes, cytochrome P450 family members, NQO1, Ya subunit of glutathione *S*-transferase (GST), δ-aminolevulinic acid synthase, UDP-glucuronosyltransferase and others (Beischlag et al., 2008; Furue et al., 2017). There are studies reporting the cross-talk between AhR and Nrf2 signaling pathway (Miao et al., 2005; Korashy and El-Kadi, 2006; Tsuji et al., 2012; Dietrich, 2016). It was shown that Nrf2 promoter contains three XRE-like elements (XREL) located at position -712 (XREL1), +755 (XREL2) and +850 (XREL3). The activity of 2, 3, 7, 8-tetrachlorodibenzo-p-dioxin (TCDD)-induced Nrf2 mRNA was abolished in transient AhR-silenced cell line tao (Miao et al., 2005). Besides, activation of AhR nuclear translocation by ketoconazole (KCZ) has shown to upregulate cytochrome P450 family 1 Subfamily A Member 1 (CYP1A1) expression. Meanwhile, it also induced the Nrf2 nuclear translocation, resulting in upregulation of NQO1 expression (Tsuji et al., 2012). These evidences suggest that activation of Nrf2 can be regulated by AhR-ARNT pathway.

##### Epigenetic regulation of Nrf2

Epigenetics modifications including DNA methylation, histone modification, and microRNAs (miRNAs) expressions are involved in Nrf2 regulation (Guo et al., 2015). For instance, the expression of Nrf2 and its downstream gene NQO1 were lower in transgenic adenocarcinoma of mouse prostate (TRAMP) C1 cells (Yu et al., 2010; Zhang et al., 2013). Treatment with DNA methyltransferases (DNMTs) inhibitor 5-aza-2′-deoxycytidine (5-Aza) and histone deacetylase (HDAC) inhibitor trichostatin A (TSA) has shown to restore the epigenetically silenced Nrf2 gene and increase NQO1 expression, which helps to prevent prostate cancer progression in TRAMP mice and protects against Alzheimer’s development in a mouse neuroblastoma N2a cellular model (Yu et al., 2010; Cao et al., 2016). In addition, TSA also increased Nrf2-regulated HO-1, NQO1, glutamate-cysteine ligase catalytic (GCLC) in neuron cultures and brain tissue by promoting Nrf2 dissociation from Keap1 and nuclear Nrf2 translocation (Wang et al., 2012).

MiRNAs are short, single-stranded, small non-coding RNA molecules of approximately 18–25 nucleotides long. It also has been implicated in regulation of Nrf2 at the post-transcriptional level (Lujambio and Lowe, 2012). Recent study showed that increased miR-200a expression leads to Keap1 degradation and Nrf2 protein stabilization, thereby protecting OB-6 osteoblastic cells from dexamethasone-induced oxidative stress and apoptosis (Zhao et al., 2017). A similar relationship was observed between miR-141 and Nrf2/Keap1 pathway in hepatocellular carcinoma cells. Increased levels of miR-141 expression in hepatocellular carcinoma cells such as HepG2, SMMC-7721, and HuH7 cell lines has shown to downregulate Keap1 expression via the Keap1 3′ untranslated region (3′ UTR), resulting in transcriptional activation of Nrf2-dependent HO-1 gene (Shi et al., 2015). Interestingly, an inverse correlation between miRNAs and Nrf2 has also been reported. Downregulation of miRNAs such as miR-93, miR-142-5p and miR-218 exhibited protective effects against cerebral ischemic injury and high glucose (HG)-induced apoptosis through upregulation of Nrf2/ARE signaling pathway (Wang et al., 2016, 2017; Liu et al., 2017).

##### Autophagy

Autophagy, a bulk-lysosomal degradation process that is responsible for the clearance of aggresomes and abnormal organelles, can enhance cell survival under stress condition. The functional role of autophagy adaptor proteins, also known as sequestosome-1 (p62/SQSTM1) in regulating Nrf2 and its downstream target genes has been elucidated (Bryan et al., 2013; Kapuy et al., 2018). Under oxidative stress condition, p62 is phosphorylated, which increases its binding affinity to Keap1. The binding of p62 to Keap1 results in the dissociation of Nrf2 from Keap1, thereby promoting Nrf2 stabilization and subsequent activation of downstream target genes. This is indicated in previous research showing that knockdown of p62 significantly promoted the accumulation of Keap1, thus enhanced Nrf2 degradation (Sun et al., 2016). In addition, depletion of SQSTM1 significantly doubled the half-life of Keap1 and lead to simultaneous decrease in Nrf2 protein and mRNA levels (Copple et al., 2010). Mammalian target of rapamycin complex 1 (mTORC1)-induced S351 phosphorylation in the Keap1-interacting region (KIR) motif of p62 markedly increase binding affinity of p62 for Keap1, thereby increasing the transcriptional activation of Nrf2 target genes (Ichimura et al., 2013). This suggests that p62 can compete with Nrf2 for binding to Keap1 via KIR motif, which has a sequence similar to the ETGE motif in Nrf2 (Jain et al., 2010). Apart from mTOR kinase-induced p62 phosphorylation, several other kinases such as class III phosphoinositide 3-kinase (PI3K) vacuolar protein sorting 34 (VPS34) (Jiang et al., 2017) and TGF-β-activated kinase 1 (TAK1) (Hashimoto et al., 2016) have also been demonstrated to phosphorylate p62 and facilitate the Keap1-p62 complex interaction, thus increasing Nrf2 expression levels. Overall, these studies revealed that p62/SQSTM1 creates a positive feedback loop for enhancing Nrf2 expression.

##### Other signaling pathways

Several protein kinases including AMP-activated protein kinase (AMPK), casein kinase 2, mitogen-activated protein kinase cascades (MAPKs): JUN-N-terminal kinase (JNK), p38 kinase, extracellular-signal-regulated kinase (ERK), GSK3β, phosphatidylinositol 3-kinase (PI3K/AKT) and pancreatic endoplasmic reticulum kinase (PERK) have been implicated in Nrf2/Keap1 interaction. Phosphorylation of the serine (Ser), threonine (Thr), and tyrosine (Tyr) residues could lead to enhanced Nrf2 stability, nuclear accumulation, and subsequent transactivation activity. Phosphorylation at Thr 172 and Ser 550 by AMPK (Zimmermann et al., 2015; Joo et al., 2016) and at Ser40 by casein kinase 2 (Apopa et al., 2008) could induce Nrf2 accumulation for ARE-driven gene transactivation. Furthermore, studies have shown that MAPKs signaling pathways have a role in the Nrf2 regulation. Recent research reported that Andrographolide, a labdane diterpenoid exerts a potential therapeutic effect against neuroinflammatory diseases through upregulation of Nrf2/HO-1 expression in astrocytes via p38 MAPK and ERK-dependent pathways (Wong et al., 2016). Similarly, it was shown that activation of p38 MAPK/Nrf2 pathway is required to induce the expression of HO-1 induction by fungal β-glucan-containing particles (β-GPs) (Ishida et al., 2018). Stimulation of p38 MAPK by anisomycin was found to phosphorylate Nrf2 protein, which promotes the interaction of Nrf2 with Keap1, thereby inhibiting nuclear translocation of Nrf2 (Keum et al., 2006). In addition, GSK3β has been reported to negatively regulate Nrf2 activity. Inhibition of GSK-3β can increase the nuclear accumulation of Nrf2 and antioxidant response in hepatocytes as well as rat with cerebral ischemia-reperfusion (Jiang Y. et al., 2015; Chen et al., 2016). Moreover, PI3K/AKT and PERK also have been reported positively regulate Nrf2 activation (Cullinan and Diehl, 2004; Zou et al., 2013).

### Role of Nrf2 in Cardiovascular Diseases

A growing body of evidence showed that Nrf2 and its downstream target genes protect against CVD development, including oxidative stress-induced endothelial dysfunction and atherosclerosis. Endothelial dysfunction marks the early stages of atherosclerosis where oxidative stress enhances endothelium cell permeability, LDL oxidation, monocyte adherence, platelet activation, vascular inflammation, as well as proliferation and infiltration of vascular smooth muscle cells (VSMCs) from media to arterial intima (Hadi et al., 2005; Grover-Páez and Zavalza-Gómez, 2009). Transplantation of Nrf2-deficient bone marrow cells in LDLR^-/-^ mice model showed a reduction in the expression levels of antioxidant enzymes [NAD(P)H dehydrogenase, NQO1, catalase and GPX1], increased macrophage migration, production of pro-inflammatory cytokines, and atherosclerotic lesions (Collins et al., 2012). Meanwhile, silencing of Nrf2 in U937 monocytic cells led to an elevation of pro-inflammatory cytokines including interleukin-1β (IL-1β), interleukin-6 (IL-6), tumor necrosis factor alpha (TNF-α), monocyte chemotactic protein-1 (MCP-1), and endoplasmic reticulum (ER) stress markers expression (Song et al., 2015). Further evidence showed that overexpression of Nrf2 in VSMCs of rabbit model showed increased expression of antioxidant enzymes (HO-1 and NQO1) and inhibition of VSMCs proliferation and vascular inflammation (Levonen et al., 2007). In addition, Nrf2 deficiency in macrophage promoted pro-inflammatory cytokines production (MCP-1, IL-6, and TNF-α) and enhanced oxidized low density lipoprotein (oxLDL) uptake, leading to foam cell formation (Ruotsalainen et al., 2013).

Interestingly, some studies reported that Nrf2-mediates pro-atherogenic effects (Sussan et al., 2008; Barajas et al., 2011; Freigang et al., 2011; Harada et al., 2012; Ruotsalainen et al., 2018). Nrf2 deficiency in ApoE^-/-^ mice developed smaller atherosclerotic plaques by reducing CD36 expression, a type of scavenger receptor which is responsible for taking up modified LDLs (Sussan et al., 2008). Moreover, a reduction in pro-inflammatory cytokine IL-1-mediated vascular inflammation was observed in Nrf2-deficient ApoE^-/-^ mice (Freigang et al., 2011). Recent studies have shown that deficiency of Nrf2 in LDLR^-/-^ and LDLR^-/-^ mice expressing apoB-100 only (LDLR^-/-^ApoB^100/100^) reduced atherosclerotic lesion development. However, Nrf2 deficiency in aged LDLR^-/-^ApoB^100/100^ mice exhibit enhanced plaque inflammation and calcification (Ruotsalainen et al., 2018). These evidences suggest that Nrf2 activation plays a dual role in CVD.

## Protective Effects of Natural Products Against Cardiovascular Diseases

Natural products derived from plants, marine organisms and animals have been a reliable source of new structural leads for treatment of various diseases. Most bioactive compounds are produced as secondary metabolites, which can be classified as phenolics, flavonoids, chalcones, terpenoids, carotenoids, anthocyanins, quinones, and others. These bioactive compounds possess a wide range of biological activities including anti-tumor, anti-inflammatory, anti-carcinogenic, anti-viral, anti-microbial, anti-diarrheal, anti-oxidant, and other activities (Manivasagan et al., 2014; Hamed et al., 2015; Wang et al., 2018). Bioactive compounds have been shown to reduce atherosclerosis formation and risk of developing CVD (Rangel-Huerta et al., 2015; Dal and Sigrist, 2016). Recently, the PREvencion con DIeta MEDiterannea (PREDIMED) trial reported that dietary polyphenols intake such as extra-virgin olive oil and nuts were associated with improved CVD risk factors and decreased inflammatory biomarkers levels in high CVD risk participants. It was shown that polyphenol intake decreased blood pressure (BP), increased plasma high density lipoprotein (HDL) and decreased the inflammatory biomarkers of CVD, including vascular cell adhesion molecule 1 (VCAM-1), intercellular adhesion molecule 1 (ICAM-1), IL-6, TNF-α as well as MCP-1 (Medina-Remón et al., 2017). Similarly, Health, Alcohol and Psychosocial factors In Eastern Europe (HAPIEE) study also reported that dietary polyphenols (phenolic acids and stilbenes) intake were found to be inversely correlated to metabolic syndrome (MetS) which is closely link to risk factors of CVD including glucose intolerance, dyslipidemia, high BP and abdominal obesity (Grosso et al., 2017). Apart from dietary supplement human trials, accumulating evidence from both *in vivo* and *in vitro* studies are also supporting the cardioprotective effects of natural products. For instance, ApoE^-/-^ mice fed with high cholesterol diet supplemented with ellagic acid (EA) exerts an atheroprotective effect by improving the antioxidant capacity, attenuated hypochlorous acid (HOCl)-induced endothelial dysfunction and increased the expression of HO-1 and Nrf2 (Ding et al., 2014). Treatment with quercetin ameliorated the high fat diet-induced MetS such as abdominal obesity, cardiovascular remodeling and liver complications in rats by increasing the expression of Nrf2, HO-1, carnitine palmitoyltransferase 1 (CPT1) and decreasing NF-κB (Panchal et al., 2012). Furthermore, cucurmin, a natural diarylheptanoids can prevent copper sulfate-induced LDL peroxidation, which is the earliest stage of atherosclerotic plaque that contributes to CVD (Mahfouz et al., 2009). Maslinic acid, a natural triterpenoid has also been shown to protect VSMCs against oxidative stress through activation of Akt/Nrf2/HO-1 pathway (Qin et al., 2014). Treatment with Tanshindiol C, a quinone derivative, attenuated oxLDL-induced macrophage foam cell formation by upregulating antioxidant peroxiredoxin 1 (Prdx1) and ATP-binding cassette transporter A1 (ABCA1) via Nrf2/Sirtuin 1 (Sirt1) signaling pathway (Yang et al., 2018b).

## Molecular Mechanisms of Cardioprotective Natural Products Targeting the Nrf2 Signaling Pathway

The cardioprotective role of natural products targeting Nrf2 signaling pathway has been widely investigated. There are multiple mechanisms that are involved in activating Nrf2, including interaction with cysteine residues on Keap1, disruption of Nrf2/Keap1 interaction, epigenetic modification and activation of protein kinases. The molecular mechanisms of natural products targeting Nrf2 signaling pathway will be discussed and summarized as schematically outlined in Table 1.

**Table 1:** Molecular mechanisms of bioactive compounds from natural products targeting Nrf2/Keap1 pathway.

Mode of action	Bioactive compounds	Classification	Sources	Model	Reference
Interaction with cysteine residues of Keap1	**Marine products**				
	Honaucin A	(S)-3-hydroxy-γ-butyrolactone and 4-chlorocrotonic acid connected via ester linkage	Cyanobacterium *Leptolyngbya crossbyana*	MCF7 breast cancer cell line	Mascuch et al., 2018

	**Plants**				
	Rutin	Flavonoid	Citrus fruits, black tea and buckwheat bran	HUVEC endothelial cells	Sthijns et al., 2017
	Withaferin A	Steroidal lactone	*Withania somnifera*	HUVEC endothelial cells, EA.hy926 endothelial cells and *in vitro* and *in silico* evaluations	Heyninck et al., 2016
	Xanthohumol	Chalcone	Hops (*Humulus lupulus*)	Rat adrenal PC12 pheochromocytoma cell line	Yao et al., 2015
	[6]-Shogaol	Phenylpropanoid	Ginger	HCT-116 colorectal carcinoma cell line	Chen et al., 2014
	Sulforaphane	Isothiocyanate	Broccoli	*In vitro* protein/chemical interaction (Keap1/sulforaphane)	Hu et al., 2011
	Falcarindiol	Polyacetylene	Parsley and carrots	HEK293 embryonic kidney cell line	Ohnuma et al., 2010
	Carnosic acid	Diterpene	Rosemary from *Rosmarinus officinalis*	Rat adrenal PC12h pheochromocytoma cell line and COS7 fibroblast-like cell line	Satoh et al., 2008
Disruption of Nrf2/Keap1 interaction	**Plants**				
	Khayandirobilide A	Andirobin-type limonoid	*Khaya senegalensis*	RAW 264.7 macrophage cell line and BV-2 microglia cells line	Zhou et al., 2018
	Epigallocatechin gallate	Catechin	Tea	THP-1 monocytic cell line and mice	Jiang et al., 2012; Sun et al., 2017
	Ethyl acetate extract	N/A	*Salvia miltiorrhiza*	Mouse mesangial cell (MMC) line SV40-MES-13 and mice	An et al., 2017
	Carexanes	Stilbenoid	*Carex distachya* Desf.	AGS gastric epithelial cell line	Buommino et al., 2017
	α-Linolenic acid	Polyunsaturated fatty acid	Canola, soybean, wild berries, perilla, and walnut	Rats	Yu et al., 2013

Epigenetic modulation	**Marine products**				
	Fucoxanthin	Carotenoid	Microalgae and seaweeds	HepG2 immortalized and human hepatoma cell line and JB6 P+ epidermal cells	Yang et al., 2018c
	**Plants**				
	Sulforaphane	Isothiocyanate	Broccoli	N2a neuroblastoma cell line	Zhao F. et al., 2018
	Corosolic acid	Pentacyclic triterpene acid	*Schisandra chinensis, Eriobotrya japonica, Lagerstroemia speciosa* L., *Orthosiphon stamineus* and *Weigela subsessilis*	Transgenic cell line of C57BL/6 mice (TRAMP-C1 cells)	Jie et al., 2018
	Dioscin	Steroid saponin	*Dioscorea nipponica* Makino	H9c2 embryonic cardiomyocyte cell line	Zhao L. et al., 2018
	Taxifolin	Flavanonol	*Pseudotsuga taxifolia, Taxus chinensis, Cedrus deodara* and *Pinus roxburghii*	HepG2 immortalized and human hepatoma cell line and JB6 P+ epidermal cells	Kuang et al., 2017
	Reserpine	Indole alkaloid	*Rauvolfia verticillata*	HepG2-C8 immortalized and human hepatoma cell line and JB6 P+ epidermal cells	Hong et al., 2016
	Quercetin	Flavonol	Red kidney bean, caper, radish, onion	Mice	Liu et al., 2015
	Z-Ligustilide	Phthalides	Radix Angelicae Sinensis	Transgenic cell line of C57BL/6 mice (TRAMP C1 cells)	Su et al., 2013
	Curcumin	Diarylheptanoid	Turmeric	Mice and rats	Khor et al., 2011; Muta et al., 2016

ERK phosphorylation	**Marine products**				
	Astaxanthin	Carotenoid	Red-colored aquatic organisms	HUVEC endothelial cells	Niu et al., 2018

	**Plants**				
	Methyleugenol	Phenylpropanoid	Clove, lemon grass, anise and laurel leaf oils	RAW 264.7 and J774A.1 macrophage cell lines	Zhou et al., 2017
	Dihydromyricetin	Flavanonol	Vine tea	HUVEC endothelial cells	Luo et al., 2017
	Sodium tanshinone IIA sulfonate	Water-soluble derivative of tanshinone IIA	*Salvia miltiorrhiza* Bunge (Danshen)	Rats	Wei et al., 2013
AMPK/GSK3β phosphorylation	**Plants**				
	Methyleugenol	Phenylpropanoid	Clove, lemon grass, anise and laurel leaf oils	RAW 264.7 and J774A.1 macrophage cell lines	Zhou et al., 2017
	Butin	Flavanone	*Dalbergia odorifera*	Mice and H9c2 embryonic cardiomyocyte cell line	Duan et al., 2017
	Betulin	Triterpene	Birch tree bark	RAW 264.7 macrophage cell line and mice	Ci et al., 2017
	Xanthohumol	Chalcone	Hops (*Humulus lupulus*)	Mice	Lv et al., 2017

p38 MAPK phosphorylation	**Plants**				
	Fisetin	Flavonol	Strawberries, persimmons and apples	Rat adrenal pheochromocytoma cells (PC12 cells)	Yen et al., 2017

PI3K/AKT phosphorylation	**Plants**				
	Dihydromyricetin	Flavanonol	Vine tea	HUVEC endothelial cells	Luo et al., 2017
	Paeonol and danshensu combination	Polyphenol	*Cortex Moutan* and *Radix Salvia miltiorrhiza*	Rats	Li et al., 2016
	Punicalagin	Phenolic	*Punica granatum* L.	Mouse macrophage cells (RAW 264.7 cells)	Xu et al., 2015
	3-Caffeoyl, 4-dihydrocaffeoyl quinic acid	Chlorogenic acid derivative	*Salicornia herbacea*	Hepa1c1c7 c hepatoma cell line	Hwang et al., 2009

### Interaction With Keap1 Cysteine Residues

Keap1 is a cysteine rich adaptor protein. Human Keap1 have a total of 27 cysteine residues which can be modified by oxidants and electrophiles. Among the cysteine residues in human Keap1, Cys 151, 273 and 288 are highly reactive and play an essential role for repression of Nrf2/ARE activation (Saito et al., 2016). Studies suggest that Keap1 cysteine residues modification by bioactive compounds derived from natural products are involved in activating Nrf2 antioxidant defense system. These bioactive compounds are reported to modify Keap1 cysteine residues via oxidation, alkylation or thiol disulfide interchange. A recent report investigating the effects of rutin, a flavonoid abundantly present in citrus fruit demonstrated that it protects against H_2_O_2_-induced oxidative stress in human umbilical vein endothelial cells (HUVECs). The study showed that rutin can target the Cys 151 of Keap1, and form an adduct with Keap1 which results in Nrf2 activation and upregulation of glutamate cysteine ligase, a glutathione biosynthesis rate-limiting enzyme which plays an important role in the endogenous antioxidant system (Sthijns et al., 2017). Honaucin A, an anti-inflammatory compound isolated from marine filamentous cyanobacterium *Leptolyngbya crossbyana* has been reported to induce alkylation of Keap1 cysteine thiols and thereby activating Nrf2/ARE pathway (Mascuch et al., 2018). Similarly, treatment with withaferin A, a steroidal lactone significantly increased expression of HO-1 in HUVECs and EA.hy926 endothelial cells by enhancing nuclear translocation of Nrf2. Analysis of the *in vitro* and *in silico* model suggested that withaferin A can interact with Cys 151, Cys 319, Cys 434, Cys 489, and Cys 613 (Heyninck et al., 2016). Apart from that, a number of bioactive compounds including xanthohumol, sulforaphane, falcarindiol, carnosic acid and (6)-shogaol have also been showed to interact with cysteine residues on Keap1, thereby stimulating its dissociation from Nrf2 and promoting Nrf2 nuclear accumulation which induces antioxidant proteins and phase II detoxification enzymes (Satoh et al., 2008; Ohnuma et al., 2010; Hu et al., 2011; Chen et al., 2014; Yao et al., 2015). The activation of Nrf2 and stimulation of downstream antioxidants and phase II detoxification enzymes expression suggest that these natural bioactive compounds represent a potential therapeutic source for prevention and treatment of CVDs.

### Disruption of Nrf2/Keap1 Interaction

Apart from modification of Keap1 cysteine residues, studies have demonstrated that bioactive compounds from natural products can disrupt the Nrf2/Keap1 interaction, thereby promoting Nrf2 nuclear translocation. For instance, EGCG is a well-known Nrf2 activator that promotes the dissociation of Nrf2/Keap1 and activate ARE genes transcription, thereby inhibiting TNF-α-induced NF-κB activation in human THP-1 cells (Jiang et al., 2012). Similar study also found that ECGC protects mice model against diabetic nephropathy through inhibiting the function of Keap1 by forming hydrogen bonds with specific residues such as Ser 508, Ser 555, Ser 602, Tyr 525, Tyr 572, Gln 530, and Arg 483 (Sun et al., 2017). Besides, α-linolenic acid (ALA) has been found to protect against DOX-induced cardiotoxicity by exerting anti-oxidative and anti-apoptosis properties in rat model. The underlying mechanism is associated with the enhancement of antioxidant defense system through Nrf2/Keap1 pathway by promoting the degradation of Keap1 and thus facilitating nuclear translocation of Nrf2, as well as activation of AKT/ERK pathway (Yu et al., 2013). Similarly, khayandirobilide A (KLA) also exhibits anti-inflammatory effect via elevated expression of HO-1 by inducing Keap1 autophagic degradation and thus facilitating Nrf2 nuclear translocation (Zhou et al., 2018).

### Epigenetic Modification

Epigenetic mechanisms have been reported to be associated with the pathogenesis of CVD. Epigenomics study in atherosclerotic human aorta demonstrated a genome-wide increase in DNA methylation during the onset and progression of atherosclerosis (Zaina et al., 2014). Studies have demonstrated that DNA demethylation and histone (de)acetylation can trigger or increase Nrf2 expression. Natural compounds derived from plants (such as sulforaphane, corosolic acid, taxifolin, reserpine, quercetin, Z-ligustilide and curcumin) and marine constituents (fucoxanthin) have been shown to activate Nrf2 signaling through epigenetic regulation (Khor et al., 2011; Su et al., 2013; Liu et al., 2015; Hong et al., 2016; Muta et al., 2016; Kuang et al., 2017; Jie et al., 2018; Yang et al., 2018c; Zhao F. et al., 2018). Quercetin, a natural flavonoid, significantly inhibited nickel-induced inflammation in mouse liver by decreasing Nrf2 DNA methylation and inhibiting the p38 MAPK signaling pathway (Liu et al., 2015). In addition, some natural compounds are involved in regulating miRNAs expression, which in turn activate Nrf2 pathway. For instance, treatment with dioscin, a natural steroid saponin markedly decreased the expression level of miRNA-140-5p, and subsequently activates Nrf2 and silent information regulator factor 2-related enzyme 2 (Sirt2) in cardiac H9c2 cells, thereby upregulating downstream target genes such as HO-1, NQO1, GST, GCLM, and forkhead box O3 (FOXO3a) (Zhao L. et al., 2018). However, the mechanism underlying epigenetic pathway responsible for the cardioprotective effects of natural product remains to be elucidated.

### Protein Kinases Modulation

Protein kinases such as ERK, AMPK, GSK3β, p38 MAPK, and PI3K/AKT can mediate Nrf2 phosphorylation which enhance Nrf2 stability, thereby promoting nuclear Nrf2 translocation and transactivation activity (Nguyen et al., 2003; Bryan et al., 2013). Several natural compounds, including sodium tanshinone IIA sulfonate (STS), dihydromyricetin (DMY), methyleugenol (MLG), and astaxanthin have been shown to phosphorylate ERK, and upregulate Nrf2 expression (Wei et al., 2013; Luo et al., 2017; Zhou et al., 2017; Niu et al., 2018). STS, a water-soluble derivative of tanshione IIA, was found to protect against isoproterenol (ISO)-induced myocardial infarction (MI) in rat model. Pre-treatment with STS has dramatically increased ERK phosphorylation, and subsequently enhanced the expressions of Nrf2 and HO-1 (Wei et al., 2013). DMY has also been shown to ameliorate oxLDL-induced oxidative injury in HUVECs through activation of Akt/ERK/Nrf2/HO-1 (Luo et al., 2017). Similarly, MLG protects against t-BHP-triggered cytotoxicity and attenuated ROS generation by inducing antioxidant enzymes expression and ERK phosphorylation in RAW 264.7 and J774A.1 murine macrophage cell lines. Interestingly, MLG has also shown to phosphorylate AMPK and GSK3β, which leads to upregulation of Nrf2 and its downstream target genes (Zhou et al., 2017). Besides targeting ERK pathway, other cardioprotective natural products such as butin, xanthohumol, botulin, fisetin, paeonol and danshensu combination (PDSS), punicalagin and 3-caffeoyl, 4-dihydrocaffeoyl quinic acid can activate Nrf2 through phosphorylation AMPK, GSK3, p38 MAPK, and PI3K/AKT.

## The Cardioprotective Mechanisms of Natural Products Regulating Nrf2-Associated Cellular Processes in Cardiovascular Diseases

Apart from targeting the Nrf2/Keap1 interaction, some cardioprotective natural products were found to regulate Nrf2-associated cellular processes. The mechanisms of natural products in regulating Nrf2-associated cellular processes were summarized and tabulated in Table 2.

**Table 2:** Molecular mechanisms of bioactive compounds from natural products targeting Nrf2-associated cellular processes.

Nrf2-associated cellular processes in CVD	Bioactive compounds	Classification	Sources	Mechanisms of action	Model	Reference
Regulation of ROS and glutathione levels	Curcumin	Diarylheptanoid	Turmeric	† Nrf2, † HO-1, GSH, GRD, GST and SOD, † GSH/GSSG ratio	Primary cultures of rats cerebellar granule neurons	Gonzlez-Reyes et al., 2013
	5-*O*-caffeoylquinic acid	Chlorogenic acid	Coffee	† Nrf2, † γ-GCL, HO-1 and GSTA1	HT29 colon carcinoma cell line	Boettler et al., 2011
	Azafrin	Carotenoid	Dried root of *Centranthera grandiflora*	† Nrf2, † mRNA expression levels of HO-1, NQO1, GCLC, GCLM, Trx1 and GST	HEK293 embryonic kidney and H9c2 embryonic cardiomyocyte cell lines	Yang et al., 2018a
	Triptolide	Diterpenoid epoxide	*Tripterygium wilfordii* Hook F	† Nrf2, ↓ TNF-α, IL-1β, IL-6 and MDA, † HO-1, SOD, GSH and GPx	Rats	Yu et al., 2016
Crosstalk with NF-κB inflammatory signaling pathway	Ligustilide	Phthalide	*Cnidii Rhizoma* and *Angelicae Gigantis Radix*	† Nrf2, † HO-1, † intracellular NO synthesis, ↓ TNF-α-ROS, ↓ NF-κB, ↓ ICAM-1, VCAM-1 and E-selectin	HUVEC endothelial cells and HL-60 leukemia cells	Choi et al., 2018
	(-)-7(S)-hydroxymatairesinol	Lignan	Norway spruce (*Picea abies*)	† Nrf2, † superoxide dismutase and HO-1, ↓ phosphorylation of ERK and Akt, ↓p65, ↓ NF-κB, ↓ TNF-α-induced VCAM-1, IL-6 and iNOS, ↓ ROS	Rat aortic endothelial cells (RAECs)	Yang et al., 2017
	Baicalein	Flavone	*Scutellaria baicalensis* and *Scutellaria lateriflora*	† Nrf2, † HO-1, ↓ IκBα phosphorylation and p65, ↓ NF-κB, ↓ TBARS, iNOS and nitrites	Mice	Sahu et al., 2016
	Cyanidin-3-*O*-glucoside	Anthocyanins	Food plants rich in anthocyanins	† Nrf2, † HO-1 and NQO-1, ↓ NF-κB, ↓ E-selectin and VCAM-1	HUVEC endothelial cells	Fratantonio et al., 2015
	Curcumin	Diarylheptanoid	Turmeric	† Nrf2, † HO-1, GCLC, and NQO-1, ↓ NF-κB, ↓ TNF-α, IL-1β and IL-6, ↓ caspase-3, Bax and † Bcl2, ↓ TGF-β	H9c2 embryonic cardiomyocyte cell line	Zeng et al., 2015
	Antrodia salmonea	Fungus	Rotten trunk of *Cunninghamia konishii*	† Nrf2, † HO-1 and γ-GCLC, ↓ NF-κB, ↓ I-κB degradation and phosphorylation of IKKα, ↓ MMP-9 and ICAM-1	EA.hy926 endothelial cells and U937 leukemic monocyte lymphoma cell line	Yang et al., 2014
	Sulforaphane, benzyl isothiocyanate and phenethyl isocyanate	Isothiocyanates	Cruciferous vegetables	† Nrf2,† HO-1, GCLC and GCLM, ↓ ROS, ↓ NF-κB, ↓ ICAM-1, VCAM-1 and E-selectin	HUVEC endothelial cells and HL-60 leukemia cell line	Huang et al., 2013
Regulation of cholesterol uptake and efflux	Tanshinone IIA	Phenanthrenequinone	*Salvia miltiorrhiz*a Bunge (Danshen)	† Nrf2, † HO-1, ↓ SR-A, † ABCA1 and ABCG1	THP-1 monocytic cell line and Mice	Liu et al., 2014
	Tanshindiol C	Phenanthrenequinone	Root of *Salvia miltiorrhiz*a Bge.	† Nrf2 and Sirt1, †Prdx1, †ABCA1	Primary cultures of rats cerebellar granule neurons	Yang et al., 2018b
	Epigallocatechin-3-gallate	Catechin	Tea	† Nrf2, ↓TNF-α-induced NF-κB activation, †ABCA1	HT29 colon carcinoma cell line	Jiang et al., 2012
	4-*O*-methylhonokiol	Phenolic	*Magnolia officinalis*	† Nrf2 and Akt2, ↓ CD36	HEK293 embryonic kidney and H9c2 embryonic cardiomyocyte cell lines	Zhang et al., 2015
	Oleanolic acid	Pentacyclic triterpenoid	*Fructus Ligustrum lucidum* and *Forsythiae fructus*	† Nrf2, †HO-1, ↓LOX-1 and NADPH oxidase subunits	Rats	Jiang Q. et al., 2015
	Salidroside	Tyrosol glucoside	*Rhodiola rosea*	↓ Phosphorylation of JNK, ERK, p38 MAPK,† Akt, † Nrf2, ↓ LOX-1, †ABCA1	HUVEC endothelial cells and HL-60 leukemia cell line	Ni et al., 2017

*An upward-pointing arrow (†) indicates increase; a downward-pointing arrow (↓) indicates decrease.*

### Regulation of ROS and Glutathione Levels

Glutathione (GSH) is a sulfhydryl group tripeptide composed of glutamate, cysteine and glycine. It plays an important role in cellular redox homeostasis. Several studies suggested that GSH can protect cells against oxidative stress due to its capability in interacting with antioxidant enzymes. Examples of GSH-linked defense enzymes include GSH peroxidase (GPX), GST, glutathione reductase (GRD), thioredoxins (Trx), glutaredoxin (GRX2), and PRX (Ribas et al., 2014; Ye et al., 2015). Glutathione is present mainly in reduced form and it is only oxidized into glutathione disulfide (GSSG) in the presence of oxidative stress. Thus, the ratio of GSH and GSSG within the cells is often used as the index for intracellular oxidative stress. Furthermore, clinical evidences have shown an association between GSH level and CVD. Low GSH level was observed in patients with the most severe cases of heart failure. Patients with cardiac diseases showed 21% depletion in blood GSH than healthy controls (Damy et al., 2009). Chronic depletion of myocardial GSH levels was reported in GCLM-deficient mice after transverse aortic constriction-induced pressure overload, and this condition leads to increased left ventricular dilation, myocardial fibrosis, and dysfunction (Watanabe et al., 2013).

Recent studies have reported the role of natural products at inducing glutathione-linked enzymes via Nrf2 signaling pathway. Azafrin, a natural carotenoid, have shown to induce Nrf2 downstream target genes such as HO-1, GCLC, GCLM, Trx1 and GST (Yang et al., 2018a). This is in consistent with another study showing that pre-treatment with triptolide, a diterpenoid epoxide protected Wistar rat from myocardial ischemia/reperfusion injuries by suppressing the production of pro-inflammatory cytokines (TNF-α, IL-1β, and IL-6) and inducing Nrf2-regulated antioxidant enzymes (SOD, GSH, GPx, and HO-1) (Yu et al., 2016). Besides, coffee constituent 5-*O*-caffeoylquinic acid (CGA) has also been shown to enhance Nrf2 nuclear translocation and increase the transcriptional expression of γ-GCL, HO-1 and GSTA1 (Boettler et al., 2011). Curcumin on the other hand has been found to attenuate hemin-induced ROS production and increase the ratio of GSH/GSSG in primary cultures of rat cerebellar granule neurons (CGNs). Furthermore, it also increased the cytoprotective enzymes such as HO-1, GR, GST, and SOD via inducing Nrf2 nuclear translocation (Gonzlez-Reyes et al., 2013).

### Crosstalk With NF-κB Inflammatory Signaling Pathway

The transcription factor NF-κB plays a crucial role in regulating innate immunity and inflammatory responses, and it is also involved in the pathogenesis of atherosclerotic plaques formation (Maracle et al., 2018). Recent studies showed that there are potential crosstalk between NF-κB and Nrf2. Nrf2 deficient mice were found to have increased NF-κB activation, inflammatory cytokines TNF-α, IL-1β, and IL-6 production, and ICAM-1 expression in brain after traumatic brain injury compared to wild-type mice (Jin et al., 2008). Evidence suggests that Nrf2/Keap1 pathway can inactivate NF-κB activity through ubiquitin-mediated degradation of IKKβ (Lee et al., 2009). Besides, it has been reported that p65, a canonical NF-κB subunit antagonized the transcriptional activity of Nrf2 by depriving Nrf2 transcriptional co-activator CBP (Liu et al., 2008). These data suggest that there is a crosstalk between Nrf2 and NF-κB in regulating the transcription of its downstream target proteins.

Several natural compounds have been reported to protect against CVDs by targeting both Nrf2 and NF-κB signaling pathways. For instance, Ligustilide, a phthalide compound inhibited VCAM-1, ICAM-1 and E-selectin expression by suppressing the NF-κB activation as well as inducing Nrf2-mediated HO-1 expression in TNF-α-stimulated HUVECs (Choi et al., 2018). Similarly, bioactive compounds such as cyanidin-3-*O*-glucoside and isothiocyanates have been reported to counteract the pathogenesis of endothelial dysfunction, including upregulation of Nrf2-dependent antioxidant response elements (HO-1, GCLC and GCLM) and downregulation of adhesion molecules (ICAM-1, VCAM-1, and E-selectin) via inhibition of NF-κB activation (Huang et al., 2013; Fratantonio et al., 2015). Furthermore, *Antrodia salmonea*, a medicinal fungal species exerts an anti-angiogenic and anti-atherogenic activity in human vascular endothelial cell line (EA.hy 926) and human leukemic monocyte lymphoma cell line (U937). It significantly suppressed TNF-α-induced matrix metalloproteinase-9 (MMP-9) and ICAM-1 expression via suppressing I-κB degradation and I-κB kinase phosphorylation, as well as upregulating the expression of HO-1 and γ-GCLC through Nrf2 signaling pathway (Yang et al., 2014).

### Regulation of Cholesterol Uptake and Efflux (Foam Cell Formation)

Macrophage foam cell formation represents the early hallmarks of atherosclerosis lesion formation. Foam cells formation is closely associated with abnormal cholesterol metabolism that results from imbalanced cholesterol uptake and efflux. Macrophages may take up modified LDL via scavenger receptors or through the pinocytosis process. Scavenger receptors such as scavenger receptors class A (SR-A), scavenger receptor class B (CD36), and lectin-type oxidized LDL receptor (LOX-1) have been implicated in the pathogenesis of atherosclerosis (Kunjathoor et al., 2002; Schaeffer et al., 2009). Several studies suggested that SR-A and CD36 exert pro-atherogenic properties due to their ability to interact with modified LDL, thereby contributing to the foam cell formation. Silencing SR-A or CD36 alone in LDLR^-/-^ ApoB100 mice was shown to profoundly protect against atherosclerosis (Mäkinen et al., 2010). Besides, studies have shown that cholesterol efflux transporters such as scavenger receptor class B type 1 (SR-B1), ABCA1 and ABCG1 promote efflux of free cholesterol to apolipoproteinA-1 (apoA-1) and HDL. Deficiency of either one or both efflux transporters (ABCA1 or ABCG1) have shown to enhance lipopolysaccharide (LPS)-induced inflammatory gene expression, reduce aortic endothelial NO synthase (eNOS), increase monocyte adhesion and infiltration into atherosclerotic plaque (Westerterp et al., 2016). Similarly, double-knockout ABCA1 and ABCG1 mice administered with a high cholesterol diet exhibited extensive infiltration of macrophage foam cells in the myocardium and spleen and have shown accelerated progression in atherosclerosis (Yvan-Charvet et al., 2007; Westerterp et al., 2013).

The role of Nrf2 in the transcriptional regulation of these scavenger receptors and cholesterol efflux transporters has been established. Many recent studies have reported the role of natural compounds in modulating cholesterol uptake and efflux receptors by targeting the Nrf2 signaling pathway. Oleanolic acid (OA), a natural pentacyclic triterpenoid, exerts anti-atherosclerotic effect in quail models and HUVECs where it was shown to inhibit oxLDL-induced LOX-1 and NADPH oxidase subunits expression while increasing the expression of Nrf2 and HO-1 (Jiang Q. et al., 2015). Furthermore, salidroside protects against foam cells formation by upregulating ABCA1 and downregulation of LOX-1 via activation of MAPK/Akt/Nrf2 pathways (Ni et al., 2017). This is consistent with another study which showed that Tanshinone IIA (Tan) inhibit atherosclerotic plaque formation in ApoE^-/-^ mice and it is suggested that Tan regulates cholesterol metabolism by reducing the expression of SR-A, while further enhancing ABCA1 and ABCG1 expression in human THP-1 cells via activation of ERK/Nrf2/HO-1 pathway (Liu et al., 2014). Other plant constituents (as shown in Table 2) such as Tanshindiol C, epigallocatechin-3-gallate, and 4-*O*-methylhonokiol (MH) can regulate SR-A and CD36 receptors via Nrf2 activation and its downstream effects.

## Recent Insights of the Clinical Investigation of Nrf2-Activators

Nrf2 is a potential therapeutic target for the treatment of multiple sclerosis, diabetes mellitus, Parkinson’s disease, cancer, and others diseases. Several Nrf2 activators were currently being tested in human clinical trials. For instance, bardoxolone methyl is an OA derivative tested in the Phase II/III clinical trials for the treatment of Alport syndrome, a genetic disorder characterized by glomerulonephritis, eye abnormalities, and hearing loss (Chin et al., 2018; Gross et al., 2018). Bardoxolone methyl activates Nrf2 by disrupting the Nrf2/Keap1 interaction and inhibits IKKβ kinase activity (Wang et al., 2014). It was also reported for its beneficial effect in Phase III clinical trial for type 2 diabetes and stage 4 chronic kidney disease patients, where it was shown to improve the estimated glomerular filtration rate (GFR) (Chin et al., 2018). It is suggested that bardoxolone methyl increases GFR by restoring endothelial dysfunction and reducing angiotensin II-induced glomerular mesangial cell contraction (Aminzadeh et al., 2013; Ding et al., 2013). In addition, Protandim^®^, a nutritional supplement containing five natural Nrf2 activators such as bacosides, silymarin, withanolides, ECGC and curcumin has been shown to reduce oxidative stress and increase antioxidant enzymes SOD and catalase expression (Nelson et al., 2006). Similarly, increased SOD expression was also observed after oral Protandim^®^ supplementation in runners (Ueberschlag et al., 2016). Considering that many studies have reported the role of natural compounds in activating Nrf2 pathway, further investigations and validations in the clinical setting may help to accelerate its development as therapeutics protecting against CVD.

## Conclusion

Transcription factor Nrf2 serves as the master regulator of cellular antioxidant defense system which has shown to protect against endothelial dysfunction, foam cells formation and atherosclerotic lesion development. Compelling evidences in this paper have demonstrated that a wide range of bioactive compounds derived from natural sources activate Nrf2/Keap1 signaling and protect against CVD development. These studies suggest that bioactive compounds may serve as new therapeutic strategies targeting CVD via Nrf2 pathway.

## Author Contributions

This writing was performed by BO. BG and K-GC provided vital guidance and insight to the work. WY did some literature review and amended the review. K-GC, BG, and WY contributed to the funding of the project. The project was conceptualized by WY and BG.

## Conflict of Interest Statement

The authors declare that the research was conducted in the absence of any commercial or financial relationships that could be construed as a potential conflict of interest.
